# Efficient Parallel Implementation of Active Appearance Model Fitting Algorithm on GPU

**DOI:** 10.1155/2014/528080

**Published:** 2014-03-02

**Authors:** Jinwei Wang, Xirong Ma, Yuanping Zhu, Jizhou Sun

**Affiliations:** ^1^School of Computer Science and Technology, Tianjin University, Tianjin 300072, China; ^2^College of Computer and Information Engineering, Tianjin Normal University, Tianjin 300387, China

## Abstract

The active appearance model (AAM) is one of the most powerful model-based object detecting and tracking methods which has been widely used in various situations. However, the high-dimensional texture representation causes very time-consuming computations, which makes the AAM difficult to apply to real-time systems. The emergence of modern graphics processing units (GPUs) that feature a many-core, fine-grained parallel architecture provides new and promising solutions to overcome the computational challenge. In this paper, we propose an efficient parallel implementation of the AAM fitting algorithm on GPUs. Our design idea is fine grain parallelism in which we distribute the texture data of the AAM, in pixels, to thousands of parallel GPU threads for processing, which makes the algorithm fit better into the GPU architecture. We implement our algorithm using the compute unified device architecture (CUDA) on the Nvidia's GTX 650 GPU, which has the latest Kepler architecture. To compare the performance of our algorithm with different data sizes, we built sixteen face AAM models of different dimensional textures. The experiment results show that our parallel AAM fitting algorithm can achieve real-time performance for videos even on very high-dimensional textures.

## 1. Introduction

Detecting and tracking moving deformable objects in a video sequence is a complex and difficult task and has been a very important part of many applications, such as human computer interaction [1], automated surveillance [2], and emotion recognition [3]. This task allows us to determine the state of objects and helps us analyze their behaviors.

The active appearance model (AAM) [4], first proposed by Cootes et al. [5], is one of the most powerful model-based object detecting and tracking algorithms. It is a nonlinear, generative, and parametric model and can be traced back to the active contour model (or “snakes,” [6]) and the active shape model (ASM) [7]. Particularly, the AAM decouples and models the shape and the texture of the deformable object to generate a variety of instant photos realistically. Therefore, the AAM has been widely used in various situations [8–10]. The most frequent application of AAMs to date has been face modeling and tracking [11].

Although the AAM possesses powerful modeling and efficient fitting ability, the high computational complexity caused by the high-dimensional texture representation limits its application in many conditions, for example, real-time systems. To make the AAM more applicable to practical applications, additional effort must be spent to accelerate the computation of the AAM. Therefore, several improvements are proposed to achieve this aim. Some methods are proposed to reduce the dimension of the texture, such as the Haar wavelet [12], the wedgelet-based regression tree [13], and the local sampling [14]. However, these methods improve efficiency at the expense of decreasing accuracy or losing detail information. From another perspective, researchers [15, 16] suggest reformulating the AAM in an analytic way to speed up the model fitting. A famous method is the inverse compositional image alignment (ICIA) [17] algorithm that avoids updating texture parameters every frame and is a very fast-fitting algorithm for the AAM. However, the limitation of this algorithm is that it cannot be applied to the AAMs that parameterize the shape and appearance variation with a single set of parameters.

Currently, as a general-purpose and high performance parallel hardware, modern graphics processing units (GPUs) are being developed continuously. This creates new opportunities for these complex and time-consuming algorithms to be greatly accelerated and applied in real-time systems. GPUs are dedicated hardwares for manipulating computer graphics. Due to the vast computing demand for real-time and high-definition 3D graphics, modern GPUs have evolved into highly parallel processors that feature a many-core, fine-grained parallel architecture that can handle thousands of threads running concurrently and that is very suitable for the computation of high-dimensional data with low coupling. However, the complex architecture makes designing parallel algorithms on GPUs a difficult task. All the details should be carefully considered.

In this paper, we conduct systematic research for designing AAM fitting algorithms for GPUs. We use Nvidia's compute unified device architecture (CUDA) [18, 19] as the programming model, which has been used to accelerate a large number of applications [20–22]. By analyzing the CPU serial algorithm, we find that the most time-consuming part of this algorithm is the computation of the appearance error between the model and frame. Therefore, we focus on this part and propose an efficient parallel implementation of the AAM fitting algorithm on GPUs. Our design idea is fine grain parallelism in which we distribute the texture data of the AAM, in pixels, to thousands of parallel GPU threads for processing, which makes the algorithm fit better into the GPU architecture. We target the Nvidia's GTX 650 GPU, which has the latest Kepler architecture and is designed from the outset for scientific computations, to implement our algorithm and build sixteen face AAM models of different dimensional textures to measure the performance of our algorithm. The experiment results show that our parallel AAM fitting algorithm can achieve real-time frame rates for video sequences even on very high-dimensional textures that require a large amount of computations.

The remainder of the paper is organized as follows.  Section 2 describes the basic AAM algorithm.  Section 3 describes the GPU architecture and the CUDA programming model.  Section 4 presents the design of parallel AAM fitting algorithms for GPUs.  Section 5 describes the experiments and results.  Section 6 concludes the paper and presents some future work.

## 2. Active Appearance Model

In this section, we describe the basic AAM algorithm, which has two procedures: modeling and fitting, which mostly follow the work of [4, 5].

### 2.1. AAM Modeling

**Figure 1:**
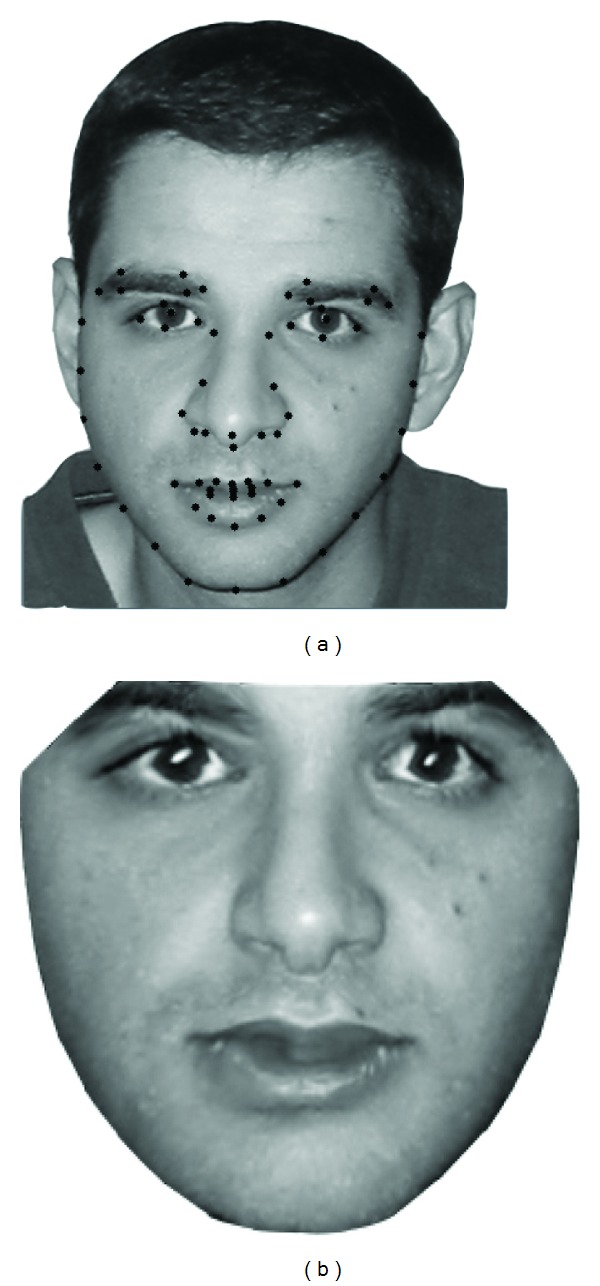
(a) Shape and (b) the shape-free patches.

The AAM requires a combination of shape and texture models to form a combined appearance model. The shape is a vector that is described by a set of concatenating position elements of the labeled landmarks, while the texture describes the measure of pixels, which is usually represented by intensities or colors. For modeling, a training set of labeled images, in which key landmark points are marked on each example object, is required. A sample-labeled image is shown in Figure 1(a).

As described in [4, 5], the shape and the texture are modeled, respectively. The landmark points on a single object describe the shape of that object. In two-dimensional cases, the shape can be represented by a vector *s* consisting of the coordinates of *n* landmark points:

(1) $$\begin{array}{c} s={\left(x_{1},x_{2},\ldots ,x_{n},y_{1},y_{2},\ldots ,y_{n}\right)}^{T}. \end{array}$$

The shapes are aligned into a common coordinate frame by applying the Procrustes analysis [23] and reduced in dimensionality from 2*n* values to a more manageable size with the principal component analysis (PCA). Then, any example can be approximated using the following:

(2) $$\begin{array}{c} s=s_{0}+P_{s}\cdot b_{s}, \end{array}$$

where *s*_0_ denotes the mean shape that can be calculated from the training set of labeled images, *P*_*s*_ = {*s*_*i*_} is the matrix that consists of a set of orthonormal base vectors *s*_*i*_ and describes the mode of variations derived from the training set, and *b*_*s*_ is a vector that defines the set of parameters of the shape model.

To build a statistical model of the texture, each example image in the training set is warped by applying a triangulation algorithm so that its control points match the mean shape to produce “shape-free patches.” A sample of the shape-free patches is shown in Figure 1(b).

On the basis of the shape-free patches, the texture can be raster scanned into a vector, *g*_*im*_. To minimize the effect of global lighting variation, the texture is linearly normalized by applying offsetting (changing brightness) and scaling (changing the contrast) of the entire shape-free patches, and the normalized texture is given by *g* = (*g*_*im*_ − *β*1)/*α*, where *α* is the scaling factor and *β* is the offset. The values of *α* and *β* are chosen to best match the vector to the normalized mean. Let *g*_0_ be the mean of the normalized data, scaled, and offset so that the sum of the elements is zero and the variance of the elements is unity. The values of *α* and *β* required to normalize *g*_*im*_ are then given by *α* = *g*_*im*_ − *g*_0_ and *β* = (*g*_*im*_ − 1)/*n*, where *n* is the size of the vector. The mean of the normalized data is obtained by an iteration process as the normalization is defined in terms of the mean.

In a similar manner, as with the shape models, PCA is ultimately applied to the normalized data, resulting in

(3) $$\begin{array}{c} g=g_{0}+P_{g}\cdot b_{g}, \end{array}$$

where *g*_0_ denotes the mean texture, *P*_*g*_ = {*g*_*j*_} is the matrix consisting of a set of orthonormal base vectors, *g*_*j*_, and describes the modes of variations derived from the training set, and *b*_*g*_ is a vector defining the parameters of the texture model.

Finally, the coupled relationship between the shape and the texture is analyzed by PCA, and the appearance model is created, which can be described as follows:

(4) $$\begin{array}{c} s⟵s_{0}+Q_{s}c,\\ g⟵g_{0}+Q_{g}c, \end{array}$$

where *c* is a vector of appearance parameters controlling both the shape and the texture, *s*_0_ is the mean shape, *g*_0_ is the mean texture in a mean-shaped patch, and *Q*_*s*_ and *Q*_*g*_ are matrices describing the modes of variation derived from the training set.

### 2.2. AAM Fitting

The purpose of AAM modeling is fitting the model to new images, which is the main concern of this paper. AAM fitting is essential for finding the most accurate parameters of the model for an object. However, the task is an unconstrained optimization problem, which is difficult to solve.

A shape in the image frame, *S*, can be generated by applying a suitable transformation to the points, *s*: *S* ← *St*(*s*). Typically, *St* will be a similarity transformation described by a scaling, *s*, an in-plane rotation, *θ*, and a translation (*t*_*x*_, *t*_*y*_). For linearity, we represent the scaling and rotation as (*s*_*x*_, *s*_*y*_), where *s*_*x*_ = (*s*cos⁡⁡*θ* − 1) and *s*_*y*_ = *s*sin⁡*θ*. The pose parameter can be described by vector *t* = (*s*_*x*_,*s*_*y*_,*t*_*x*_,*t*_*y*_)^*T*^. The texture in the image frame is generated by applying a scaling and offset to the intensities, *g*_*im*_ = *T*_*u*_(*g*), where *u* is the vector of transformation parameters. The appearance model parameters, *c*, and the shape transformation parameters, *t*, define the positions of the model points in the image frame, *S*, which gives the shape of the image patch to be represented by the model. During fitting, the pixels in this region of the image are sampled into a vector *g*_*im*_ and projected into the texture model frame, *g*_*s*_ ← *T* ^−1^(*g*_*im*_). The current model texture is given by *g*_*m*_ ← *g*_0_ + *Q*_*g*_ *c*. The current error between model and image (measured in the normalized texture frame) is thus

(5) $$\begin{array}{c} r\left(p\right)=g_{s}-g_{m}, \end{array}$$

where *p* are the parameters of the model, *p* ^*T*^ = (*c* ^*T*^|*t* ^*T*^|*u* ^*T*^). The error can be calculated simply by summing the squares of the elements of *r*, *E*(*p*) = *r* ^*T*^ *r*.

Suppose that during fitting the current residual is *r*. The model should choose the appropriate Δ*p*, which is a small variance of *p*, to minimize ||*r*(*p*+Δ*p*)||^2^. According to [4] the Δ*p* can be computed as follows:

(6) $$\begin{array}{c} \Delta p=-Rr\left(p\right), \end{array}$$

where *R* is the linear relationship (or gradient matrix) between Δ*p* and *r*. In the basic AAM, *R* is assumed to be fixed and is estimated once from a training set by multivariate linear regression techniques.

On the basis of (5) and (6), the AAM fitting is actually an optimization problem, which can be solved by an iterative procedure. Given a current estimate of the model parameters, *c*, the pose *t*, the texture transformation *u*, and the image sample at the current estimate, *g*_*im*_, the whole iterative procedure is shown in Algorithm 1.

This procedure is repeated until no improvement is made to the error, *E*, and convergence is assumed. The complexity of the algorithm is *O*(*n*_pixels_ × *n*_modes_). Essentially, each iteration involves sampling *n*_pixels_ pixel points from the image and then multiplying by the *n*_pixels_ × *n*_modes_ matrix.

## 3. GPU Architecture and CUDA Model

Before presenting the parallel AAM fitting algorithm for GPUs, we need to introduce some basic concepts about GPUs and the CUDA.

At the hardware level, a GPU device is a collection of multiprocessor units called streaming multiprocessors (SM), each of which consists of a set of processor cores called streaming processors (SP). Every SM has a single-instruction, multiple-thread (SIMT) architecture, which means that, at any given clock cycle, each SP executes the same instruction but operates on different data. Currently, the most recent GPU architecture of Nvidia (Kepler) features up to 192 SPs and a maximum of 2048 resident threads per SM [24].

The CUDA is a heterogeneous computing model that involves both the CPU and the GPU. In the CUDA model [18], a single source program includes host codes running on the CPU and kernel codes running on the GPU. Here, the GPU is regarded as a coprocessor that is capable of executing a large number of threads in parallel. Compute-intensive and data-parallel procedures are implemented as kernel codes to be executed on the GPU. Each kernel is executed as a batch of threads organized as a grid of thread blocks, and each block of threads is executed concurrently on one SM. Threads in a block can perform barrier synchronization, but there is no native synchronization mechanism for different thread blocks, except by terminating the kernel. The blocks, in turn, are divided into sets of threads called warps, each of which is formed by 32 parallel threads, which are the scheduling units of each SM. When a warp stalls, the SM can schedule another warp to execute. A warp executes one instruction at a time so that full efficiency can only be achieved when all 32 threads in the warp have the same execution path.

The CUDA model threads may access data from multiple memory spaces during their execution. Each thread has private local memory to keep its own data. All threads can access the same global memory. Both local and global memories typically come from standard off-chip memory. Each block has shared on-chip memory that is visible to all threads and has the same lifetime as the block. Two read-only memory spaces are also accessible by all threads: the constant and texture memory spaces. The properties of the different types of memory have been summarized in [25]. Because it is on chip, shared memory has a much higher bandwidth and lower latency than local and global memory. If no conflict exists, access to the shared memory only requires one or two clocks, which is almost as fast as the access to the register. Therefore, shared memory can be used for many different functions, for example, parallel reduction. Moreover, both constant and texture memories are cached on chip to provide higher effective bandwidth by reducing requests to off-chip memory. Generally, some lookup tables or invariants can be stored in constant or texture memory to accelerate access to data [19].

## 4. Design of Parallel AAM Fitting Algorithm for GPUs

In this section, we conduct an analysis on the CPU serial algorithm to find the most time-consuming part. Next, we present the design idea of our parallel algorithm. Finally, we describe each part of the parallel algorithm in detail.

### 4.1. Analysis for the CPU Serial Algorithm

As indicated in [26], the first step in designing the parallel algorithm is to analyze where the hotspots exist. We should always focus on the hotspots, which should be priorly parallelized. By analyzing Algorithm 1, we find that this algorithm can be divided into three main parts.

1. Line 3 estimates *p*, the initial values of the model parameters.
2. Lines 4 and 9 compute *E*, the error between model and image.
3. Line 6 computes Δ*p*, the small variance of *p*.

**Figure 2:**
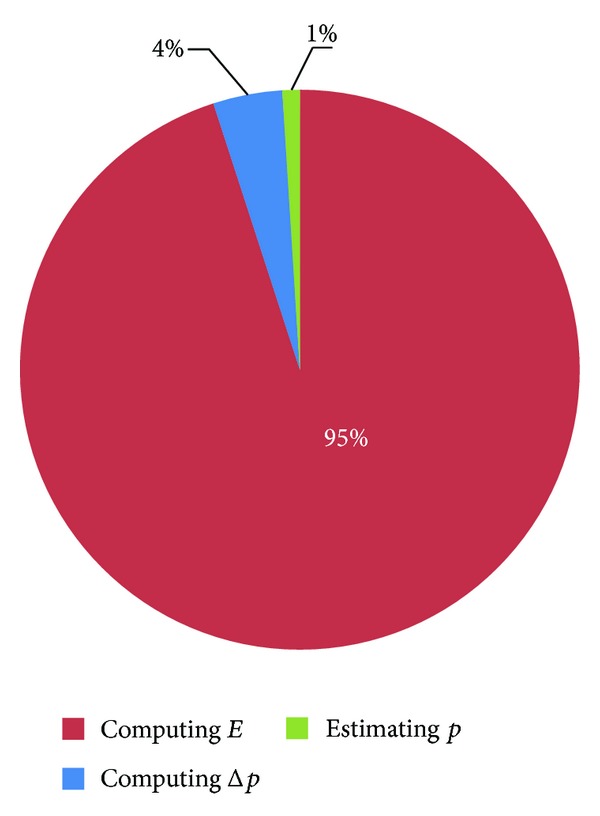
The numbers in this pie chart are the runtime percentages of each part of the AAM fitting algorithm. Different colors represent different parts of the algorithm.

Other operations, including condition, loop, and assignment statements, are small and can be ignored. We implemented Algorithm 1 in C++ and measured the runtime of the three parts on the CPU, assuming that all the operations could be processed within the main memory. The result is shown in Figure 2. Note that the runtime percentage of computing *E* is up to 95% and those of the other two parts, Δ*p* and *p*, are only 4% and 1%, respectively. Obviously, computing *E* is the hotspot of the AAM fitting algorithm that most deserves to be parallelized.

### 4.2. Design Idea of the GPU Parallel Algorithm

The applications that can be parallelized on the GPUs have features that work on a large amount of data with low coupling. Therefore, in this section, we further analyze the features of the hotspot to find the method and how to parallelize it.

The CPU-based algorithm of computing *E* is shown in Algorithm 2. This algorithm can be separated into five subprocedures.

1. Lines 1 and 2 calculate the shape of the image patch, *S*. First, compute the current shape, *s*, and then do a similarity transformation on *s* to get *S*.
2. Line 3 calculates the texture, *g*_*s*_. First, sample the textures in *S* from the image, *im*, and then project them into the texture model frame to get *g*_*s*_.
3. Lines 4 and 5 normalize *g*_*s*_. First, scale *g*_*s*_ so that the textures have zero mean and the image is unit length, and then align *g*_*s*_ to the mean texture *g*_0_.
4. Line 6 calculates the current model texture, *g*_*m*_.
5. Lines 7 and 8 calculate the *r* and final *E*.

**Figure 3:**
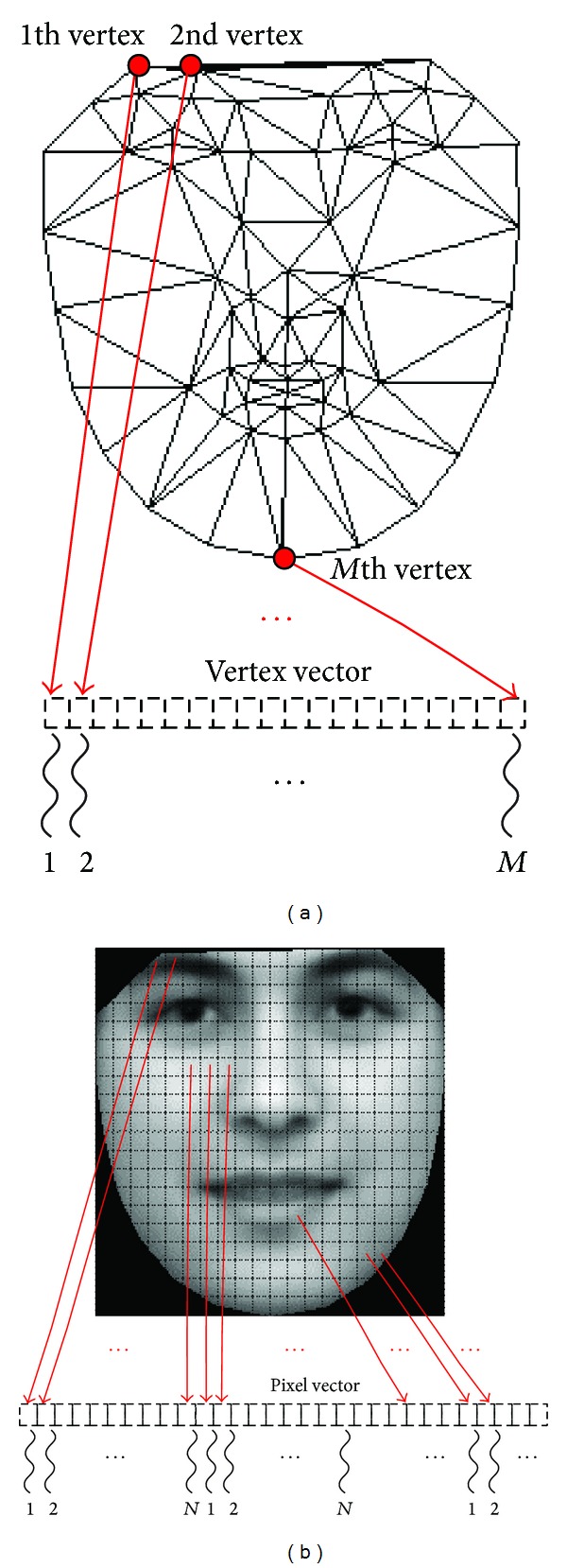
(a) The design idea of calculating the shape vector, *S*. Here, *M* is the number of vertices in *S*, which equals the number of threads involved in the calculation. (b) The design idea of the remaining four subprocedures focuses on computing *g*_*s*_ and *g*_*m*_.

In the first subprocedure, *S* is a vector consisting of the coordinates of *n* vertices. Note that the computation for each element of *S* is almost the same, which includes a multiplication between *c* and one line of matrix *Q*_*s*_, an addition with one element of vector *s*_0_, and a similarity transformation. Therefore, the design idea for parallelizing this part is to use a vertex as a unit. In practice, the number of vertices in AAM is not much more, usually from tens to hundreds. Thus, we can dispatch one thread to compute one element of *S*. This design idea is shown in Figure 3(a).

The remaining four subprocedures focus on computing *g*_*s*_ and *g*_*m*_, which are the vectors consisting of pixel intensities that describe the texture of the current frame and model. We found that these pixel intensities are mutually independent and the computing process of the intensities is almost the same. Therefore, we adopted a fine grain parallelism that divides these pixel intensities into many sets and assigns them to the parallel threads for processing. In practice, the pixels in the AAM are usually tens of thousands, and there are thousands of threads running on the GPU. Thus, each thread is dispatched to compute only several pixels, and the computational speed will be very fast. This design idea is shown in Figure 3(b). To further improve the computational speed, we make each thread calculate one pixel for every *N* pixels, where *N* is the total number of threads involved in the calculation. Therefore, the access pattern for pixel intensities will meet the criteria of coalesced memory access on the GPUs [27].

### 4.3. Details of the Parallel Algorithm

According to the idea presented in Section 4.2, we designed the GPU parallel algorithms for every part of the hotspot, computing *E*. In the following sections, we define *gridDim* as the dimension of the grid, *blockDim* as the dimension of the block, *blockId* as the current block ID, and *threadId* as the current thread ID.

#### 4.3.1. Calculating Shape *S*

The GPU parallel algorithm for calculating the shape, *S*, is shown in Algorithm 3. For computational convenience, the vector *S* is stored in the form (*x*_1_, *y*_1_, *x*_2_, *y*_2_,…, *x*_*n*_, *y*_*n*_), where (*x*_*i*_, *y*_*i*_) is the coordinate of the *i*th vertex. Line 1 computes the thread ID in the grid and also the vertex ID. Lines 2–5 compute the coordinate, (*x*, *y*), of the *i*th vertex of the current model shape, which corresponds to line 1 in Algorithm 2. Lines 6–9 conduct a similarity transformation (scaling, rotation, and translation) to the *i*th vertex, which corresponds to line 2 in Algorithm 2. Line 10 ensures that the coordinates of all vertices are in the image.

#### 4.3.2. Calculating Texture *g*_*s*_

Algorithm 4 is the GPU parallel algorithm for calculating *g*_*s*_. Line 1 computes the thread ID in the grid and also the pixel ID in the shape patch of the current frame. Line 2 computes the total number of threads in the grid. Lines 4–7 correspond to line 3 in Algorithm 2. First, line 4 receives the IDs of three vertices of the triangle that the *i*th pixel belongs to in the shape vector, *S*. Next, lines 5 and 6 conduct a piecewise affine to compute the coordinate (*x*, *y*) of the equivalent pixel in the image frame, *img*. Here, *α*, *β*, and *γ* are the coefficients that give the relative position of the pixel in the triangle. Finally, line 7 conducts a bilinear interpolation to smooth the sampled texture, *g*_*s*_, when scaling it. Lines 3–9 form a loop, which computes the pixels dispatched to this thread one by one.

#### 4.3.3. Normalizing Texture *g*_*s*_

The GPU parallel algorithm of normalizing *g*_*s*_ is shown in Algorithm 5 and corresponds to lines 4 and 5 in Algorithm 2. Algorithm 5 has four parts, which are separated by three global synchronizations underlined on lines 14, 28, and 42, because the CUDA provides no guaranteed method for global synchronization except between kernel launches. When implemented, Algorithm 5 is broken into four different kernels that are launched in order. Each global synchronization launches a new kernel. Lines 1–13 compute the average *g*_*s*_, *mean*. Lines 15–27 calculate the Euclidean norm (*norm*) of difference between *g*_*s*_ and *mean*. Lines 29–41 compute the dot product (*α*) between *g*_*s*_ and *g*_0_. Lines 43–49 scale *g*_*s*_ with *α*.

**Figure 4:**
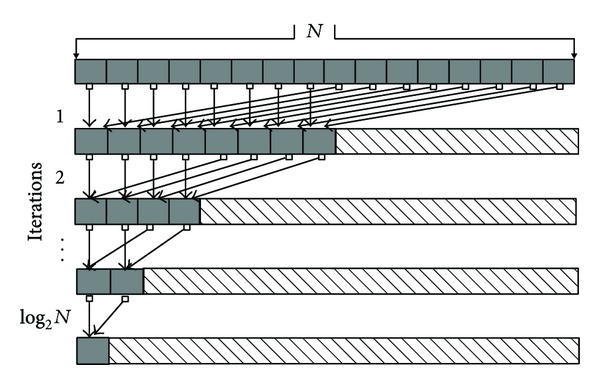
Coalesced access pattern for the elementwise operation on a vector and the subsequent parallel reduction operation per thread group in shared memory.

In Algorithm 5, we compute three sums, including the sum of elements in *g*_*s*_ for calculating *mean*, the sum of squares of elements in *g*_*s*_ for calculating *norm*, and the sum of products between the elements of *g*_*s*_ and *g*_0_ for calculating *α*. To decrease the global memory reading latency, we use a shared memory array, *SMData*, and adopt the three-level summation method. The first level is the thread level, in which each thread computes the sum of data dispatched to it and stores the result into a cell of *SMData*. The second level is the block level, in which all threads in a block conduct a parallel reduction on the *SMData *that belongs to this block to receive the partial result. The third level is the grid level, in which the thread with zero ID from each block adds the reduction result from each block to the variable *tmp* in global memory using the CUDA atomic function, atomicAdd(). The access pattern for the parallel reduction on the shared memory array,* SMData*, is illustrated in Figure 4, which is designed to avoid shared memory bank conflicts [28]. This reduction requires log⁡_2_⁡(*N*) iterations.

#### 4.3.4. Calculating Texture *g*_*m*_

Algorithm 6 shows the GPU parallel algorithm of computing *g*_*m*_, which corresponds to line 6 in Algorithm 2. Line 1 computes the thread ID in the grid and also the pixel ID in the shape of the current model. Lines 3–10 compute *g*_*m*_ by multiplying one line of matrices  *Q*_*g*_ and *c* and then adding one element of vector *g*_0_.

The structure of the parallel algorithm for computing *r* and *E* is similar to that of Algorithm 6.

#### 4.3.5. Nonhotspots of the Algorithm

When we completed the design of the parallel algorithm of the hotspot, computing *E*, we conducted a preliminary test. The results show that the performance of our parallel algorithm is very good. However, the two nonhotspots implemented as serial code on the CPU, estimating *p* and computing Δ*p*, reduced the performance of the entire fitting algorithm. Therefore, we decided to parallelize these two parts.

Estimating *p* is only executed once every frame, and its computation process is similar to that of *g*_*s*_. Therefore, we parallelize this part according to the methods described in Section 4.3.2.

Computing Δ*p* is actually a matrix-vector multiplication between matrix *R* and vector *r*. Initially, to parallelize this part, we use the SGEMV function of CUBLAS 5.0 library [19] provided by Nvidia. However, this function is not efficient, especially for very wide matrices. For example, here, the matrix *R* has tens of rows and tens of thousands of columns because the typical parallelization of SGEMV on GPUs is implemented using several threads (e.g., a warp) to perform a dot product between one row of the matrix and the vector to produce one element of the result vector. Thus, when the matrix is very wide, each thread conducts a large number of calculations and the performance will observably reduce. Therefore, we designed a novel autotuning method for matrix-vector multiplication on GPUs, where the number of threads used to compute one element of the result vector can be autotuned according to the matrix size [29]. For very wide matrices, thousands of threads are used to compute one element of the result vector. This method allows our kernel to achieve a higher performance than the SGEMV of CUBLAS 5.0.

## 5. Experiments and Results

### 5.1. Experiment Platform

Our experiments were conducted on a PC with an NVIDIA GTX650 GPU card and an AMD Athlon II X 250 CPU. The GTX 650 GPU is based on the new NVIDIA architecture: Kepler GK107. This GPU has two streaming multiprocessors, called SMX, and each SMX has 192 scalar processors or 384 processor cores. Each core performs at 1058 MHz. The memory of this GPU card is 1 GB of DDR5 DRAM with the peak bandwidth of 177.4 GB/s. GTX650 has computational capabilities of 3.0, in which the maximum number of resident threads per SMX is 2048, the maximum number of threads per block is 1024, and the maximum amount of shared memory per SMX is 48 KB. The CPU has two cores running at 3.0 GHz. The main memory is 8 GB of DDR3 DRAM with the peak bandwidth of 12.8 GB/s.

Our parallel AAM fitting algorithm is implemented for single-precision arithmetic and is compiled by the CUDA 5.0 compiler with optimization option/O2. The whole parallel algorithm includes ten kernels. Algorithms 3, 4 and 6, each corresponds to one kernel. Algorithm 5 corresponds to four kernels. Calculating *r* and *E*, estimating *p*, and computing Δ*p*, each corresponds to one kernel.

To avoid the long latency time of global memory access, prior to the fitting process, our CUDA program copies all the invariant data, including matrix *Q*_*g*_, matrix *Q*_*s*_, vector *g*_0_, and vector *s*_0_ to the texture memory using a cache mechanism and is more efficient than using global memory.

When implementing the kernel of Algorithm 3, we only use one thread block with the same size as the number of vertices in the shape of the image patch. For the other nine kernels, our experiments show that if four thread blocks are used, each with a size 1024, and if a total of 4096 threads run on the GPU simultaneously and reach 100% occupancy, then we receive the best performance. Additionally, we used the multistreams technique [18] in the CUDA to hide the transfers of frame and shape data between the host and the device.

### 5.2. Training and Test Data

We chose the FG-NET face database [30] as the training database. This database contains 1002 face images of 82 subjects from multiple races with ages ranging from 0 to 69 years. Each image in the database has 68 labeled, facial landmarks characterizing shape features.

**Figure 5:**
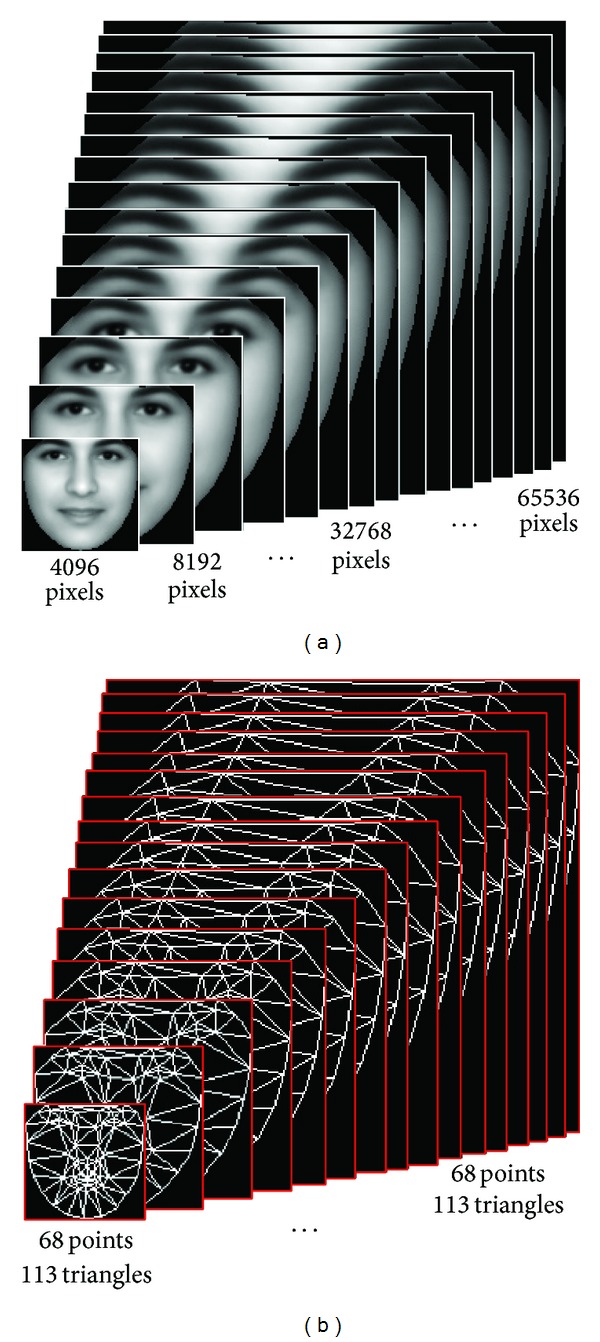
(a) Texture templates and (b) shape templates.

From the FG-NET database, we selected one image per person from the 82 different test subjects, with ages ranging from 11 to 51, as the training images (82 images in all). All training images were then converted to gray images. To compare the performance of the algorithm with different data sizes, we scaled the coordinates of the original 68 labeled landmarks of the training images and obtained 16 new training sets. After training the AAM on these training sets, we obtained 16 face models, each of which has 68 landmark points and 113 triangles. For convenience of measurement, these models are built in a single-resolution framework. The difference in these models is the pixel number, which denotes the dimension of the texture in the shape of the image patch. The *i*th models have *i* ×4096 pixels, for a total of 4096 to 65536 pixels.  Figure 5 shows the texture and shape templates of the 16 models.

To evaluate the real-time performance of the parallel AAM fitting algorithm, we chose a video database, the YouTube face database [31], as the test database. This new face database contains 3425 video clips of 1595 different people. All the clips were downloaded from YouTube at 24 frames per second. The database clips include single faces, multiple faces, faces blocked by objects (glasses, hats, other persons, etc.), and faces with changing illuminations. Because we parallelized the basic AAM fitting algorithm, which does not consider any complex situations, we selected 50 clips of a single face of different people that were not blocked by any object and that had an unchanged illumination as the test set. The shortest clip duration in this test set is 2 seconds, and the longest clip is 71 seconds.

### 5.3. Experimental Results and Performance Evaluation

For the GPU parallel algorithm, we fit each clip of the test set using the 16 models described in Section 5.2. To facilitate the performance measurement and to avoid the impact of the different data on the performance, we applied a fixed number of iterations for each frame, 10, which is about the average number of iterations measured on the test set, applying an unfixed number of iterations. We used each model to fit every clip several times, recorded the raw data, and computed the average values. Comparatively, we repeated the steps above for the CPU algorithm.

**Figure 6:**
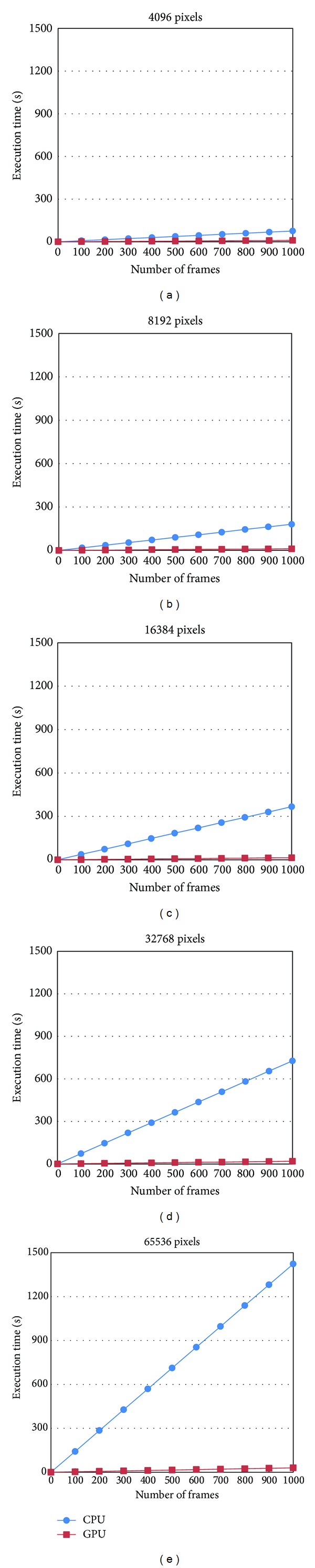
The execution times of the CPU serial algorithm and the GPU parallel algorithm increase with the number of frames on different data sizes.

In Figure 6, we plot the trends of the execution times of the CPU serial algorithm and the GPU parallel algorithm and observed an increase in the number of frames for different data sizes. Here, we selected 5 typical data sizes that increase exponentially from 4096 pixels (Figure 6(a)) to 65536 pixels (Figure 6(e)). As shown in each subfigure of Figure 6, the trends of the CPU and the GPU times are all linear, and the growth rate of the GPU times is obviously much lower than that of the CPU times with an increase in frames. From another perspective, with the growth of the data size, the line's gradient of the CPU time increases very rapidly. By comparison, the line's gradient of the GPU time increases slowly.

**Figure 7:**
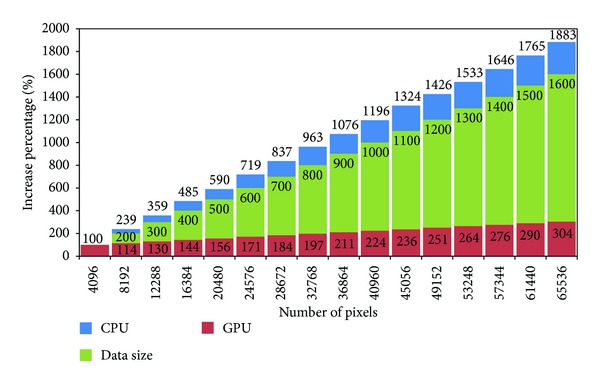
The growth speeds of the execution time of the CPU serial algorithm and the GPU parallel algorithm.

Next, we plot the growth speeds of the execution time of the CPU serial algorithm and the GPU parallel algorithm in Figure 7. We use the time of 1000 frames for the minimum data size, 4096 pixels, as the basic value and compute the percentage of the times for all data sizes and the basic value. As shown in Figure 7, when the data size changes from 4096 pixels to 65536 pixels, a 16-time increase, the CPU time increases 18.8 times. Comparatively, the GPU time increases only 3 times.

Table 1 shows the details of the time per frame and the time per iteration of each data size using a microsecond as the unit. Each value is the average number of execution times for 1000 frames. For the minimum data size, 4096 pixels, our GPU parallel algorithm takes only 9.74 milliseconds per frame, which is equivalent to 102.67 frames per second. For the median data size, 32768 pixels, the GPU parallel algorithm takes 19.15 milliseconds per frame or 52.22 frames per second. For the maximum data size, 65536 pixels, the GPU parallel algorithm takes 29.58 milliseconds per frame or 33.81 frames per second. Compared to the CPU-based method, our method is dozens of times faster. These results reveal that the proposed algorithm can efficiently accelerate the AAM fitting process and achieves the real-time speed of videos, even on very high-dimensional textures that need a large amount of computations.

**Figure 8:**
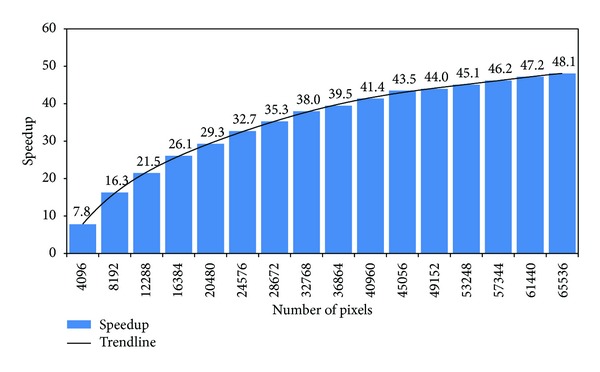
The speedup of the GPU parallel algorithm to the CPU serial algorithm for the 16 data sizes.

Finally, we researched the speedup of the GPU parallel algorithm compared to the CPU serial algorithm for the 16 data sizes. In Figure 8, we present the results, which show that, when the data size is small, for example, 4096 pixels, the speedup is very low (only 7.8). When the data size is increased, the speedup shows a growth trend, but not linear, and the growth rate becomes much slower. In our experiment, the maximum speedup appears at 48.1 for 65536 pixels, which is obviously not the maximum speedup of this problem. When we plot a trendline that is a 6th order polynomial, the trend becomes more obvious. The trendline proves that the GPU's performance is significantly improved for tasks that contain a large amount of data, which exploits the GPU's massively parallel nature. In contrast, the GPU is unsuitable for tasks with a small data volume in which some parts, such as the launch for kernels, control statements, and nonparallel parts, will take a greater percentage of the total time that is already limited.

## 6. Conclusion

In this paper, we proposed a GPU-based parallel AAM fitting algorithm. In our algorithm, we distribute the texture data, in pixels, to the thousands of parallel GPU threads for computation. This design idea makes the algorithm fit better into the GPU architecture. We implemented our algorithm on Nvidia's newest GTX650 GPU (Kepler architecture) using CUDA 5.0. To compare the performance of our algorithm with different data sizes, we built 16 face AAM models of different dimensional textures that range from 4096 pixels to 65536 pixels. The experiment results show that our parallel AAM fitting algorithm can achieve real-time performance for videos, even on very high-dimensional textures (102.67 frames per second for 4096 pixels and 33.81 frames per second for 65536 pixels). Additionally, we discovered that when the texture dimension is increased, the speedup of the GPU compared to the CPU gradually increased to 48.1. This algorithm needs a large amount of data to fully exploit the potential of the GPU.

In the future, we would like to apply our GPU-based parallel AAM fitting algorithm to applications that need the real-time detection and tracing of objects, such as automated surveillance, human computer interaction, and emotion recognition systems.

## Figures and Tables

**Algorithm 1:**
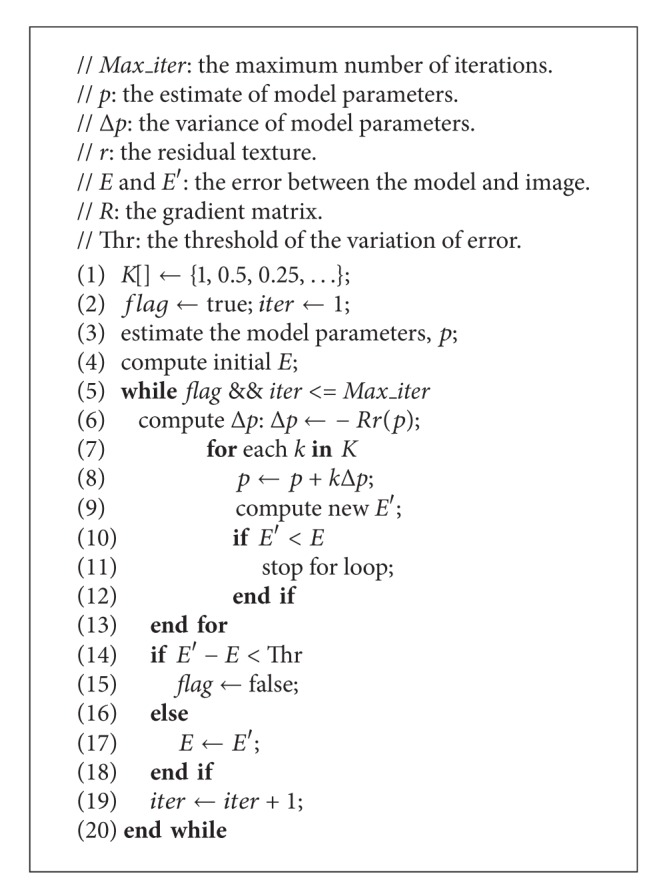
CPU-based AAM fitting algorithm.

**Algorithm 2:**
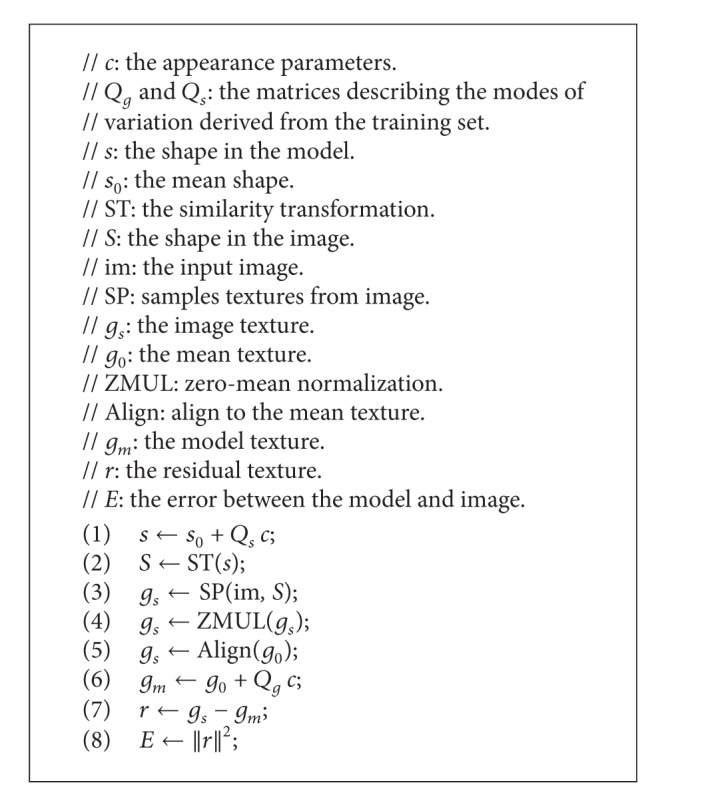
CPU-based method for computing *E*.

**Algorithm 3:**
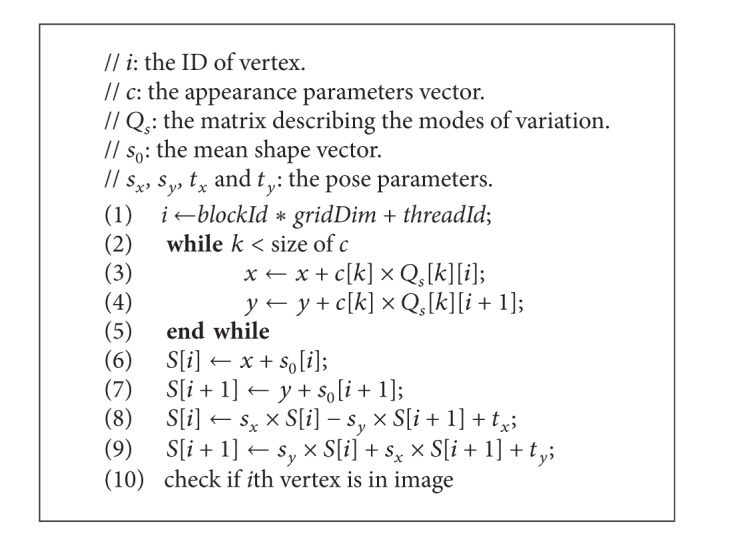
GPU-based method for calculating *S*.

**Algorithm 4:**
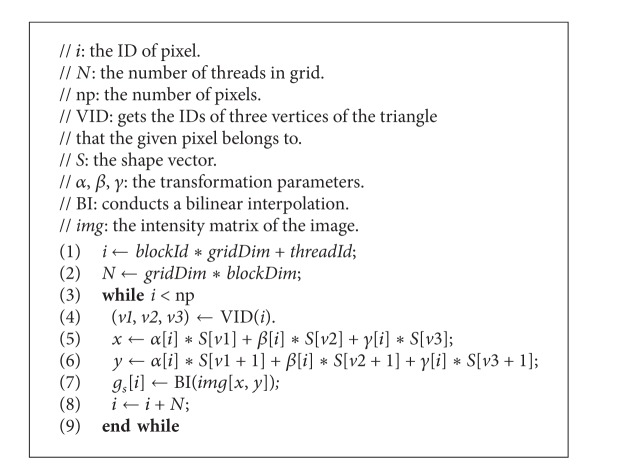
GPU-based method for calculating *g*_*s*_.

**Algorithm 5:**
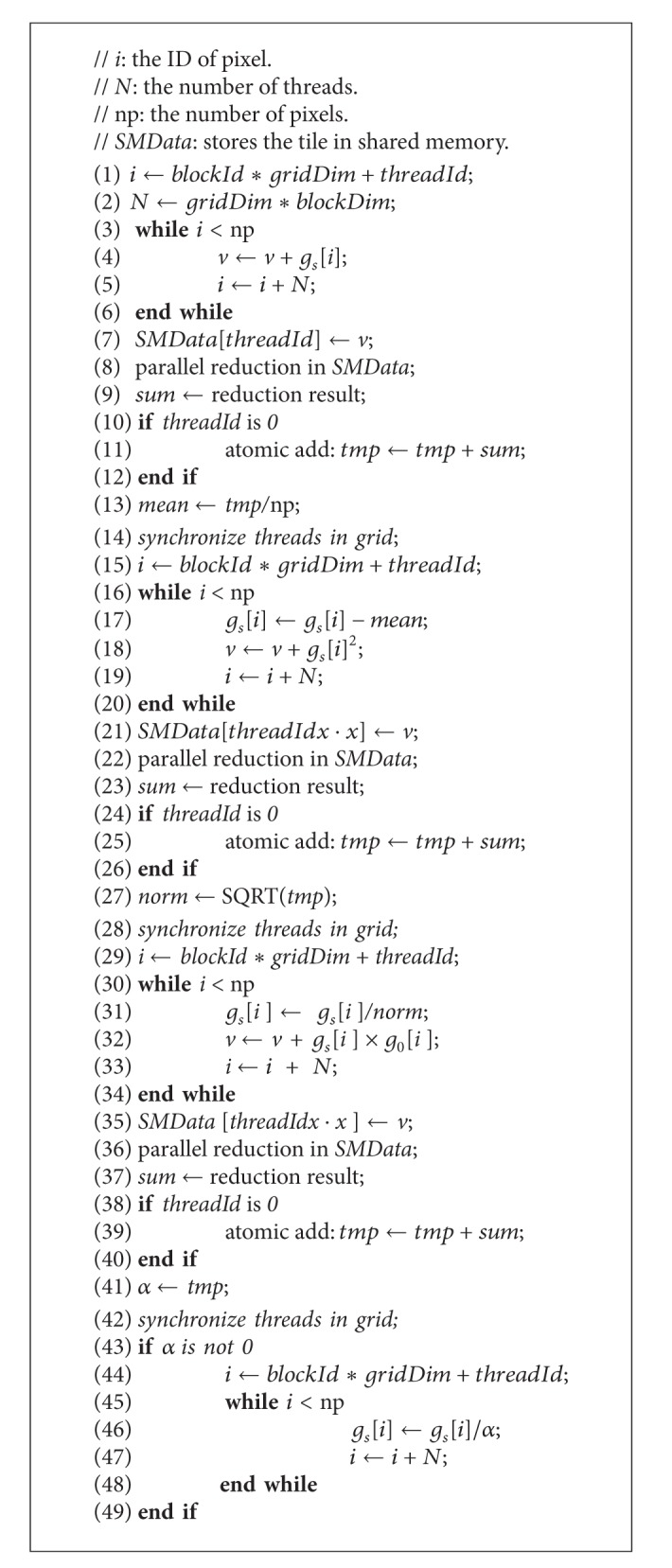
GPU-based method for normalizing *g*_*s*_.

**Algorithm 6:**
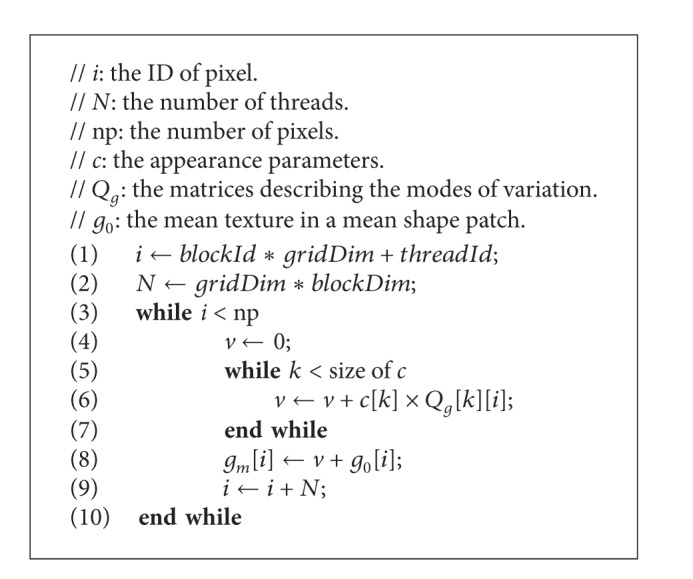
GPU-based method for calculating *g*_*m*_.

**Table 1:** The time per frame and the time per iteration of different data size.

Data size (pixels)	Time per frame (ms)		Time per iteration (ms)	
	CPU	GPU	CPU	GPU
4096	75.57	9.74	7.56	0.97
8192	180.58	11.08	18.06	1.11
12288	271.40	12.65	27.14	1.27
16384	366.81	14.06	36.68	1.41
20480	445.58	15.18	44.56	1.52
24576	543.34	16.64	54.33	1.66
28672	632.59	17.94	63.26	1.79
32768	727.48	19.15	72.75	1.92
36864	813.35	20.58	81.34	2.06
40960	903.71	21.85	90.37	2.18
45056	1000.40	23.02	100.04	2.30
49152	1077.62	24.47	107.76	2.45
53248	1158.40	26.54	115.84	2.65
57344	1243.77	26.91	124.38	2.69
61440	1333.50	28.26	133.35	2.83
65536	1423.24	29.58	142.32	2.96
